# Gut Microbiota Dysbiosis: Triggers, Consequences, Diagnostic and Therapeutic Options

**DOI:** 10.3390/microorganisms10030578

**Published:** 2022-03-07

**Authors:** Tomas Hrncir

**Affiliations:** The Institute of Microbiology, The Czech Academy of Sciences, 549 22 Novy Hradek, Czech Republic; hrncir@biomed.cas.cz

The global incidence of numerous immune-mediated, metabolic, neurodegenerative, and psychiatric diseases is steadily increasing [1,2,3]. The increased morbidity of human populations makes them more vulnerable to additional burdens, including infectious diseases. For example, the mortality rate of diabetics infected with the original variant of the SARS Co-2 virus was about twice that of the general population [4,5]. It is becoming increasingly clear that the gut microbiota plays an important role in the development of many, if not all, of these diseases [2,6,7]. Many of these diseases, including COVID-19 infection [8], are associated with alterations in gut microbiota composition and function, i.e., dysbiosis [9,10]. The most typical features of dysbiosis are a decrease in the diversity of the microbiota, a loss of beneficial microbiota, or an overgrowth of harmful microbiota. The term “gut microbiota” includes all microorganisms, i.e., not only bacteria, but also fungi, protists, archaea, and viruses that live in the gastrointestinal tract. The focus of this Special Issue was on all possible triggers of gut microbiota dysbiosis [11] and exploring ways to restore the gut microbiota, such as dietary interventions [12,13,14], probiotics, and FMT, to identify disease-associated microbiota signatures, elucidate pathogenetic mechanisms [15], find new diagnostic and prognostic markers, and develop novel and promising microbiota-based treatments.

Dysbiosis can be caused by host-specific factors such as genetic background, health status (infections, inflammation), and lifestyle habits or—more importantly—environmental factors such as diet (high sugar, low fibre), xenobiotics (antibiotics, drugs, food additives), and hygiene. 

Profound changes in the gut bacterial and fungal microbiota can be rapidly achieved by changes in macronutrients. These changes have significant physiological consequences as, for example, diets rich in simple sugars disrupt the intestinal barrier, trigger intestinal inflammation, and negatively affect host metabolism. However, in most cases, interactions between diet and microbiota are necessary for these deleterious effects, as they do not occur in the absence of the gut microbiota, i.e., in a germ-free state, and transplantation of the gut microbiota often results in transfer of the disease phenotype [16,17,18].

The effect of food additives on the gut microbiota has long been overlooked, but recently, several groups, including ours, have published data showing that some human gut microbiota are very sensitive to preservatives [10] and, also, that exposure to common food preservatives promotes overgrowth of proteobacteria [19]. Other categories of additives have also been shown to have negative effects on human health. For example, dietary emulsifiers directly alter the composition of the human gut microbiota and trigger intestinal inflammation [20]. Artificial sweeteners, such as Splenda, promote proteobacterial dysbiosis and exacerbate ileitis in SAMP1/YitFc (SAMP) mice [21]. Paradoxically, non-caloric artificial sweeteners were introduced primarily to prevent metabolic syndrome, but unfortunately, they induce dysbiosis and promote glucose intolerance in a microbiota-dependent manner, leading to the negative metabolic effects that they were intended to prevent [22].

Host-derived factors that alter the burden and composition of the gut microbiota include bactericidal fluids produced by the gastric glands and liver, i.e., gastric acid and bile, and antimicrobial molecules such as defensins, lysozymes, and antibacterial lectins (Reg3γ) produced by Paneth cells, or SIgA produced by plasma cells [23]. Many infectious diseases, even if they are not gastrointestinal, trigger dysbiosis of the gut microbiota. Interestingly, SARS-CoV-2 infection has also been associated with dysbiosis of the gut microbiota [8]. Possible mechanisms that have been proposed include leakage of SARS-CoV-2 into the gut, direct binding of the virus to angiotensin-converting enzyme 2 (ACE2) receptors expressed on the surface of enterocytes, and circulating cytokines. COVID-19-associated dysbiosis has also been linked to increased intestinal permeability, which may negatively impact disease prognosis [24].

Disruption of the gut microbiota ecosystem has many consequences, which can be divided into disruption of the gut barrier and imbalance of the host immune and metabolic systems. The integrity of the intestinal wall could be compromised by acetaldehyde produced by the microbiota from exogenous or endogenous ethanol [25], direct mucolytic activity [26], and other mechanisms. The host immune system can be modulated by microbiota-derived molecules via inflammasome signalling or Toll-like receptor (TLR) and NOD-like receptor (NLR) signalling. Another mechanism is a shift in the balance between regulatory and proinflammatory immune cells. Effects on the host metabolic system, particularly glucose and lipid metabolism, are mediated by changes in bile acid composition, production of short-chain fatty acids (SCFAs) from dietary fibre, conversion of choline to trimethylamine (TMA), and many others [27].

Improved understanding of the gut microbiota, its metabolites, and its interactions with hosts could be used to develop new diagnostic and therapeutic approaches. Invasive diagnostic methods, such as tissue biopsy, are often required to make a diagnosis, determine disease subtype, and monitor disease progression and treatment efficacy. Therefore, non-invasive and reliable markers are needed. Recent research suggests that some members of the gut microbiota and metabolites may be useful as diagnostic and prognostic markers. For example, Loomba et al. found that a panel of the gut microbiota consisting of 37 bacterial strains can be used to accurately diagnose advanced fibrosis in patients with non-alcoholic fatty liver disease (NAFLD) [28] and Lee et al. found a strong association of Veillonellaceae with liver fibrosis in non-obese NAFLD patients and propose *Veillonellaceae* as a diagnostic marker [29]. Many gastrointestinal [30,31] and metabolic diseases [32,33,34,35] are associated with decreased levels of *Faecalibacterium prausnitzii*, which is considered a beneficial microbe due to its anti-inflammatory properties [33]. Another strain, *Bacteroides vulgatus*, is elevated in advanced fibrosis [28] and severe obesity [35]. Interestingly, cirrhotic patients often have a greater amount of oral microbial strains in the gut microbiome, such as *Prevotella*, *Veillonella*, and *Streptococcus* [32], which are generally absent in healthy individuals. Alterations in the fungal microbiota, characterised by a decrease in diversity and excessive growth of *Candida* [36], are common in patients with alcoholic liver disease. The most consistent finding in patients with IBD is a decreased diversity of the microbiota, characterised mainly by a decrease in the relative abundance of the Firmicutes phylum and an increase in the Proteobacteria phylum [37,38]. At the species level, a decrease in SCFA-producing bacteria such as *Faecalibacterium prausnitzii* and *Clostridium* clusters IV, XIVa and XVIII [39] and an increase in sulphate-reducing bacteria (SRB) such as *Desulfovibrio* [40,41], an increase in mucolytic bacteria such as *Ruminococcus gnavus* and *Ruminococcus torques* [26], and an imbalance of inflammatory and anti-inflammatory species have been demonstrated [37]. 

There are several microbiota-derived metabolites that could be used as biomarkers for disease. Some examples of promising metabolites used for the detection of liver diseases are succinate, phenylacetic acid, and 3-(4-hydroxyphenyl)-lactate. The 3-(4-hydroxyphenyl)-lactate, a product of aromatic amino acid metabolism, has been associated with liver fibrosis [42]; serum levels of phenylacetic acid correlate with the severity of hepatic steatosis [43]; and succinate produced by NAFLD-associated microbes such as *Bacteroidaceae* and *Prevotella* [44] was found to be elevated in faecal, serum, and liver samples from NAFLD patients [27].

The gut microbiota could be used for therapeutic purposes on several levels. First, the entire microbiota community could be restored by faecal microbiota transplantation (FMT). Second, individual strains or collections of beneficial strains (probiotics) could be introduced into the gut microbiota to supplement missing functions, whereas harmful or undesirable strains could be removed using antibiotics, antifungals, or bacteriophages. Finally, microbial metabolic pathways could be targeted to reduce or block the production of harmful metabolites or stimulate the production of beneficial metabolites.

FMT is a highly effective and life-saving therapy for recurrent *Clostridioides difficile* infections (rCDI), with cure rates exceeding 90%. It is often recommended as initial treatment because most CDI patients recover after a single FMT treatment [45]. However, the pharmaceutical industry is pushing to classify FMT as a drug to gain market exclusivity [46,47]. FMT has also been used experimentally to treat other gastrointestinal disorders, such as ulcerative colitis [48], constipation [49], irritable bowel syndrome [50], liver diseases such as cirrhosis with encephalopathy [51] and alcoholic hepatitis [52], and also neurological diseases such as multiple sclerosis [53] and Parkinson’s disease [54].

Probiotics are very popular agents for modulating gut microbiota and host health. Probiotic microorganisms exert their effects in several ways that often work together. The main mechanisms are modulation of the immune system, resistance to colonisation, improvement of the intestinal barrier, and production of metabolites that act locally (antimicrobials, enzymes, organic acids) and remotely (neurochemicals, hormones). There are compelling data on the safety and efficacy of several probiotics, including *Lactobacillus* spp., *Bifidobacterium* spp., and *Saccharomyces* spp. Other promising candidates include *Roseburia* spp. and *Faecalibacterium* spp. [55]. Probiotics have been successfully used to treat various diseases, including necrotising colitis [56], antibiotic-induced diarrhoea [57], ulcerative colitis [58], irritable bowel syndrome [59], and acute diarrhoea [60]. There is also evidence that orally administered probiotics may be beneficial in the treatment of metabolic, cardiovascular, or neurological diseases. However, high-quality randomised controlled clinical trials are needed to better understand which strains, formulations, and dosing patterns are effective for which conditions. In addition, probiotic interventions need to be individualised based on various host factors such as diet, baseline microbiota, genetics, disease subtype, or medications.

Therapeutic approaches that aim to manipulate the microbiota to increase the production of protective metabolites or block or decrease the production of harmful metabolites are also promising. For example, 3,3-dimethyl-1-butanol, a structural analogue of choline, has been successfully used to block the microbial conversion of dietary choline to TMA [61]. Trimethylamine N-oxide (TMAO), an oxidation product of TMA, has been associated with atherosclerosis and severe cardiovascular disease [62,63]. Interestingly, a recent study suggests that TMA and not TMAO may be the main culprit due to its nephrotoxicity and cardiotoxicity [64]. Manipulating the microbiota to increase SCFA production may also be helpful, as preclinical data show that SCFA supplementation improves hepatic steatosis. For example, supplementation of tributyrin, a butyrate prodrug, protected mice from obesity, insulin resistance, and hepatic steatosis [65], while supplementation of acetate and propionate prevented diet-induced weight gain, hepatic steatosis, and insulin resistance [66]. Other promising therapeutic targets include farnesoid X receptor (FXR) and Takeda G-protein-coupled receptor 5 (TGR5) signalling pathways that modulate bile acid metabolism. For example, obeticholic acid, a potent activator of FXR, ameliorates hepatic steatosis, fibrosis, and portal hypertension in animal models of fatty liver disease [67,68,69] and treatment of adult NASH patients improved histological features [70]. In addition, NGM282, an analogue of fibroblast growth factor 19 (FGF19), i.e., a hormone that regulates bile acid synthesis and glucose homeostasis, has been shown to reduce hepatic steatosis in NASH patients [71].

In summary, there is growing evidence that the gut microbiota plays a critical role in many immune-mediated, metabolic, and neurological diseases. The most important factors that negatively affect the gut microbiota are environmental factors, particularly unhealthy diets and medications. Genetic factors probably do not play as large a role in the increase in these diseases because they are relatively stable. A dysbiotic microbiota can compromise the gut barrier, resulting in tissues and organs being flooded with molecules from the diet and microbiota that can negatively impact the host immune system and metabolism. Moreover, the negative feedback loop can actually exacerbate dysbiosis. For example, the diseased liver is unable to regulate the gut microbiota through bile acids and other microbiota-modulating factors. 

Several clinical studies have uncovered promising disease-associated microbiota signatures that could be used to detect early disease stages, monitor disease progression, and evaluate therapeutic efficacy. The diagnostic and prognostic value of microbiota signatures could be further enhanced by integration with the detection of microbiota-derived molecules in blood, urine, or faeces. 

A detailed understanding of microbiota–host interactions will also enable the development of efficient microbiota-based therapies. These include manipulating the composition of the microbiota, e.g., introducing new beneficial strains or eliminating harmful strains, or replacing the entire ecosystem by transplanting the faecal microbiota. A complementary approach is based on the use of microbial metabolites, i.e., inducing or blocking the production of specific metabolites.

Whether dysbiosis of the gut microbiota is a direct cause of disease or merely reflects disease-induced changes in the host immune and metabolic systems remains unclear, but there are several examples of changes in the gut microbiota that precede the onset of disease, such as in type I diabetes [72] and Parkinson’s disease [73], and the body of evidence in support of a central role of the gut microbiota in the pathogenesis of many immune-mediated, metabolic, and neurological diseases continues to grow.
